# Vaginal-Laparoscopic Repair (VLR) of Primary and Persistent Vesico-Vaginal Fistula: Description of a New Technique and Surgical Outcomes

**DOI:** 10.3390/jcm12051760

**Published:** 2023-02-22

**Authors:** Roberto Tozzi, Giulia Spagnol, Matteo Marchetti, Giulia Montan, Carlo Saccardi, Marco Noventa

**Affiliations:** 1 Division of Women and Children’s Health, Department of Gynaecology and Obstetrics, University of Padua, 35122 Padua, Italy; 2 Nuffield Department of Women and Reproductive Health, University of Oxford, Oxford OX3 9DU, UK

**Keywords:** vesico-vaginal fistula, laparoscopy, complications

## Abstract

The main aim of our study was to describe the surgical technique and evaluate the feasibility, efficacy and safety of a vaginal-laparoscopic repair (VLR) of iatrogenic vesico-vaginal fistulae (VVF). Between April-2009 and November-2017, we retrospectively reviewed all clinical, radiological and surgical details of surgery for benign or malignant disease and ended up with VVF. All patients were diagnosed by CT urogram, cystogram and clinical test. The surgical technique was standardised and is described here. Eighteen patients developed VVF after hysterectomy, three after caesarean section and three after hysterectomy and pelvic lymphadenectomy. Twenty-two patients had an average 3 (range 1–5) attempts at fistula repair in other hospitals. In one patient, five attempts were made. The mean size of the fistula was 2.4 cm (range 0.7–3.1 cm). A median 8 weeks (6–16) conservative management with Foley catheter failed in all patients. No conversion to laparotomy and no complication occurred at VLR. Median hospitalisation was 1.4 days (range 1–3). The latter confirmed all patients were dry and tested negative at a repeated filling test. At 36 months follow-up, all patients remained dry. In conclusion, VLR successfully repaired VVF in all patients with primary and persistent VVF. The technique was safe and effective.

## 1. Introduction

Vesico-vaginal fistula (VVF) is a condition in which the mucosa of the bladder is directly connected to the mucosa of the vagina and causes leakage of urine in the vagina. It is an uncommon occurrence, but the majority of fistula aetiologies fall into two major categories: VVF secondary to obstructed labour and secondary to iatrogenic trauma (surgery, radiation therapy, or malignancy) [1,2]. Women having VVF are continuously damp from urine leakage and sometimes suffer genital ulceration, infections, and an unpleasant smell; all these conditions can restrict their daily activities [1]. The majority of reports for VVF consisted of case series and experiences of health professionals, whereas the existing studies were not specific, with studies mostly focused on obstetric fistulas as mainstream [1].

In the developed world, VVF is most commonly associated with iatrogenic injury occurring during gynecologic surgery and hysterectomy is the operation most frequently causing a VVF with a rate of 0.08% [2].

There are many risk factors predisposing patients to VVF including prior pelvic surgery, radiation, prolonged presence of a foreign body, infection, and pelvic malignancy [3]. In the recent large series by Duong et al. that analysed 5698 hysterectomies, they found that larger uteri, longer surgeries, and more severe bladder injuries were associated with a higher risk of VVF formation [4].

The main symptom is uncontrolled discharge of urine into the vagina and the degree of urinary incontinence is typically proportional to the size of the fistula tract [1]. The timing of the diagnosis is generally 7 days to 2 weeks after the surgery, which usually is after hospital discharge. Although not life threatening, VVF is a very distressing condition for patients, with significant social and psychological repercussions. No matter the degree of incontinence, VVF can be quite debilitating with a negative impact on quality of life. In addition, it carries serious medical and social costs [1,2].

VVF should always be considered when continuous incontinence occurs following bladder catheter removal after a hysterectomy or other genitourinary procedure [1]. The workup for VVF should include: a cystoscopy to verify the distance between the VVF and the ureters’ orifices; a pelvic exam with a speculum examination to confirm the vaginal leak and to identify the exact site of the VVF by filling the bladder with normo-saline solution through the catheter; a cysto-urogram to verify the existence of the VVF, the anatomy and the size and a CT-urogram to rule out uretero-vaginal fistula [5].

Taking in account the patient’s characteristics and disease type, VVF is unlikely to heal spontaneously. The ideal timing and the surgical approach to VVF remain controversial. It is, however, very common that patients with VVF have a first attempt at conservative treatment by placing a trans-urethral and/or supra-pubic Foley catheter to void the bladder. That serves the purpose to drain the bladder and reduce the inflammation [2,3]. For smaller defects, less than 5 mm in size, conservative management could be proposed for 3 to 6 months to allow inflammation to resolve [5,6]. Spontaneous resolution of small and isolated VVF with Foley catheter drainage alone occurs in up to 39% of cases [5]. 

Surgery is the most effective corrective measure with a success rate of about 90% [3]. Several approaches have been proposed such as vaginal, abdominal, laparotomy or laparoscopy, with or without the use of flap, but none has showed to be superior. Success is often more related to the size and location of the fistula, as well as the surgeon’s experience [4]. Certainly, regardless of the surgical approach for VVF, there are some key concepts of surgical correctness that must be ensured, including adequate exposure, foreign body removal, tissue mobilization, watertight and tension-free anastomosis, multiple-layer closure, non-overlapping suture lines, urinary tract drainage, infection eradication, and meticulous hemostasis [4,5].

Transvaginal approach has been the most studied method for VVF repair because it offers different advantages over an abdominal approach including reduced operative time, hospital stay, pain, and blood loss. However, in case of deep and/or high VVF or in case of multiple/complex VVF or obese patients, the vaginal route could be extremely difficult and standard abdominal approaches may be preferred [1,4,5,6,7,8]. In these cases, minimally invasive techniques including robotic and laparoscopic surgery must definitely be considered because they can guarantee an effective surgical approach even in complex cases and a drastic reduction of intra and post-operative morbidity [7].

Finally, most studies report outcomes on primary fistula, i.e., patients whose VVF was successfully treated at the first attempt. Data on patients with persistent VVF (unsuccessfully treated by surgery and/or other methods) are lacking or undereported. 

In this study, we propose a combined vaginal and laparoscopic repair (VLR) technique that removes the entire fistulous tract rather than just separating and closing the layers. We used it to treat primary VVF and, most significantly, persistent VVF. The aim of the study is to describe the standardised surgical technique, report on the surgical outcomes, feasibility, safety and efficacy.

## 2. Materials and Methods

The study is a retrospective analysis of all patients who presented with a VVF and have been treated with VLR over a period of 8 years in three hospitals under the care of one of the surgeons (RT). We retrieved all relevant patients’ personal and clinical data, the type and size of the VVF and the cause of the VVF, with all pre-, intra-and post-operative data. Moreover, all the details about number, type and outcome of previous attempts at repair of the VVF were extracted. Patients underwent an inconsistent diagnostic path at referring institutions. Therefore, all patients underwent a repeated diagnostic under our care, which included: (1) examination to confirm the vaginal leak and identify the exact site of the VVF by filling the bladder with normo-saline solution through the catheter (2) cysto-urogram to verify the existence of the VVF, the anatomy and the size (3) CT-urogram to rule out uretero-vaginal fistula. Pre-operatively, all patients underwent cystoscopy to verify the distance between the VVF and the ureters’ orifices. All patients consented to the type of surgery and conversion to laparotomy if necessary. Although it was not compulsory, usually we waited 4 weeks between the last attempt at repair and our VLR.

### 2.1. Surgical Technique

**Figure 1:**
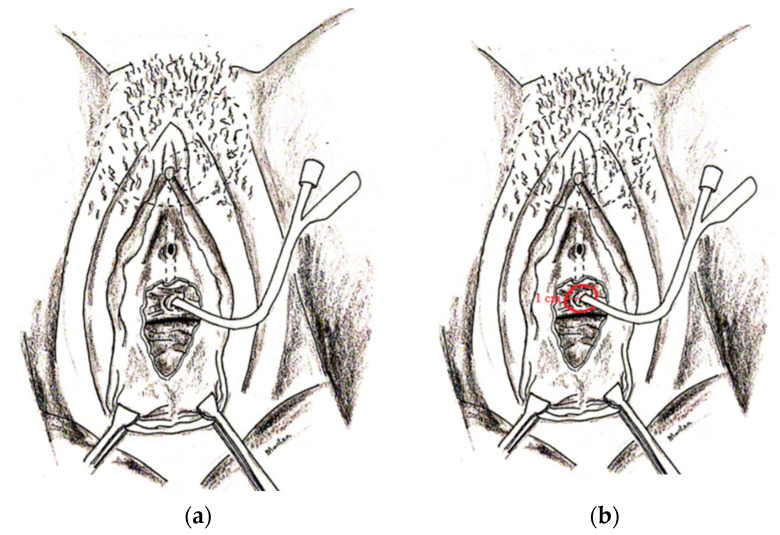
(**a**) The VVF was exposed with vaginal blades and a Foley catheter was passed through until it reached the bladder. (**b**) The vaginal part of the VVF was demarcated circumferentially by monopolar coagulation leaving at least 1 cm of healthy tissue around the edges of the VVF.

The surgery was started through the vaginal route, which was carefully disinfected. A transurethral Foley catheter size 16 Fr was placed or replaced with a new one if it was already in situ. The VVF was exposed with vaginal blades and a Foley catheter was passed through until it reached the bladder. Once in the bladder it was fixed by filling the balloon (Figure 1a).

The choice of the catheter size was the largest fitting, starting from paediatric size (6–26 Fr). At that time, the distance from the vaginal vault was accurately measured. The vaginal part of the VVF was demarcated circumferentially by monopolar coagulation leaving at least 1 cm of healthy tissue around the edges of the VVF (Figure 1b).

**Figure 2:**
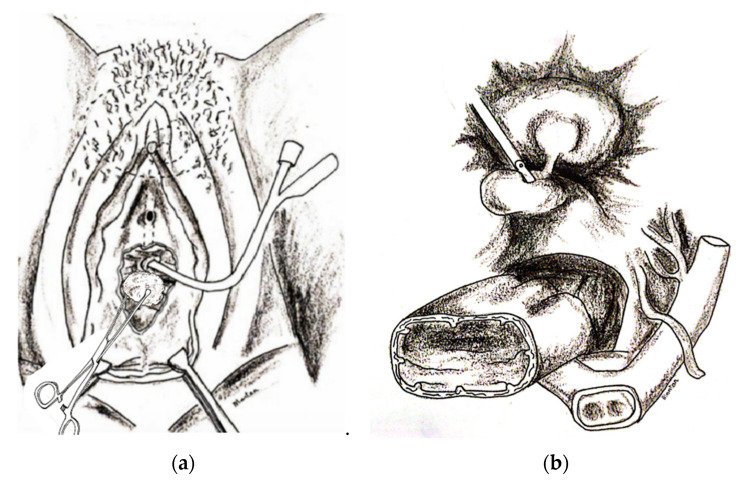
(**a**) The vaginal vault was exposed by a vaginal pressure with a gauze pad or with a bowel dilator inserted into a glove. (**b**) Laparoscopic view with exposure of VVF after careful adesiolysis and tissue dissection between bladder and vaginal vault.

After that, the laparoscopy was started with pneumoperitoneum followed by 1 × 10 mm non-disposable port (Karl Storz, Tuttlingen, Germany) in the umbilicus and 3 × 5 mm ports in the lower abdomen. After adhesiolysis, the vaginal vault was exposed by vaginal pressure exerted with use of a gauze pad or bowel dilator inserted into a glove to maintain the pneumoperitoneum (Figure 2a,b).

**Figure 3:**
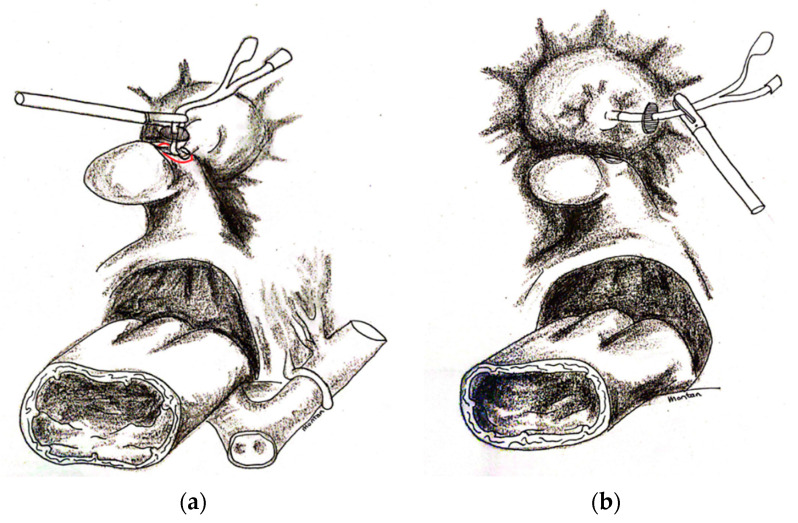
(**a**) The anterior vaginal wall was opened using monopolar to expose the distal end of the catheter with the channels. The latter part was pulled in the abdomen and the anterior vaginal wall was opened longitudinally to reach the VVF. The vaginal part of the VVF was fully resected around the catheter from the vaginal mucosa until the catheter was freed from the vagina. (**b**) At this point the Foley catheter was only held by the vesical part of the VVF.

**Figure 4:**
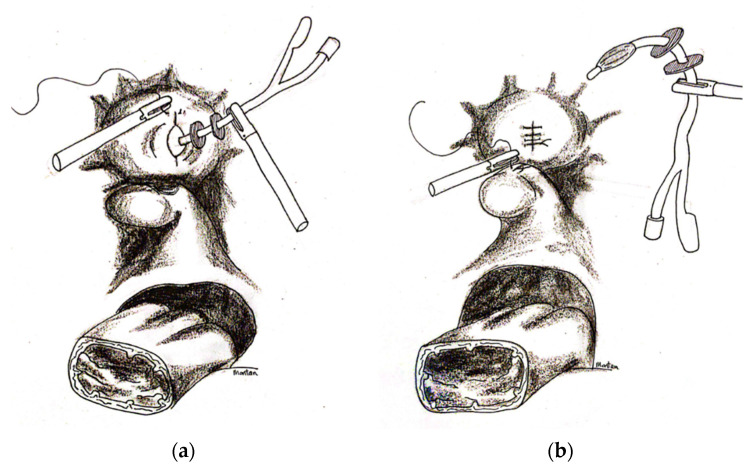
(**a**) Once obtained enough free space distally to the insertion of the catheter in the bladder, a circumferential incision of the vesical part of the VVF around the catheter followed with full resection of the tissue was performed. The bladder was then sutured in two layers helped by traction on the catheter to pinpoint the margins of the hole. (**b**) The bladder was completely sutured after deflation of the Foley. The Foley catheter was removed by deflating the balloon before the first suture was tied. Then, the vagina was sutured with intra-corporeal knots.

The anterior vaginal wall was opened using a monopolar to expose the proximal end of the Foley catheter with the channels. The latter part was pulled in the abdomen and the anterior vaginal wall was opened longitudinally to reach the VVF. The identification of the VVF was, therefore, facilitated by the lead of the Foley catheter. The vaginal part of the VVF was fully resected around the catheter from the vaginal mucosa until the catheter was freed from the vagina. At this point, the Foley catheter was only held by the vesical part of the VVF (Figure 3a,b). Thanks to the traction on the catheter, the dissection of the space below the VVF was faster. The aim was to dissect at least 3 cm distally to the VVF. Once enough free space was obtained distally to the insertion of the catheter in the bladder, a circumferential incision of the vesical part of the VVF around the catheter followed with full resection of the tissue. The bladder was then sutured in two layers, helped by traction on the catheter to pinpoint the margins of the hole. The repair was performed with interrupted intracorporeal knots using 3–0 PDS for the mucosal layer and with 2–0 Vicryl for the serosal layer run over a continuous suture (Figure 4a,b). The Foley catheter was removed by deflating the balloon before the first suture was tied. Once the first layer was completed, the bladder was inflated with normo-saline solution through the transurethral catheter exerting pressure on the suture. If any leak was identified, further sutures were placed as appropriate to eliminate any leakage. The same procedure was repeated following the second suture. The aim was for a watertight seal. The vagina was sutured with 0 PDS intra-corporeal knots on a continuous suture. No interposition graft was used. The trans-urethral Foley catheter was left in site for 3 weeks to drain the bladder. The first follow-up appointment was organised 3 weeks after the surgery. To verify the surgical outcome, the bladder integrity was tested by retrograde filling with 300 cc of normo-saline solution through the Foley catheter. Once the watertight seal was verified, the catheter was removed. After that, follow-up examination were once a year for 3 years to confirm results and prevent drop out. If new symptoms appeared, earlier checks were available.

### 2.2. Statistical Analysis

All data were analyzed using the chi-square test or Fisher’s exact test for categorical variables, and the Student’s *t*-test for continuous variables. A *p* value of 0.05 or < was considered statistically significant.

## 3. Results

Over the study period, we collected data of 24 patients with a working diagnosis of VVF. Patient demographics are shown in Table 1. Two patients were already under the team’s care. They both underwent laparoscopic hysterectomy; one for a benign and the other for a malignant condition. At the follow-up appointment, 2 weeks after the surgery, both reported vaginal leakage. After the full diagnostic flow chart, VVF was confirmed. All other patients had a working diagnosis of VVF issued at other Institutions and were referred to us for treatment of persistent VVF. They had all undergone at least 2 previous attempts at VVF repair and a maximum of 5. The initial operation causing VVF was hysterectomy plus pelvic lymphadenectomy in 3 patients (due to early stage endometrial carcinoma), simple hysterectomy in 18 patients (15 for uterine fibroids and 3 for uterine adenomyosis) and finally cesarean section in 3 patients. The number and the type of attempted repairs were extracted and reported in Table 1. Half of them also had an attempt at repair by laparotomy. 

All patients had a repeated diagnostic path to confirm diagnosis and identify as accurately as possible the anatomy of the VVF. All 24 patients were diagnosed with supra-trigonal VVF, with a mean size of 2.4 cm (range 0.7–3.1 cm) with an estimated distance of at least 2 cm away from the ureteric orifices. Due to the repeated diagnostic and waiting lists, surgery was performed 6 weeks from referral. VLR, as previously described, was successful in all 24 patients. No conversion to laparotomy occurred. We reported neither intra- nor post-operative complications based on the Clavien–Dindo classification. In none of the surgeries was an interposition graft used. All operations ended with the repairs being watertight. Surgical details are grouped in Table 2. Mean operative time was 108.3 min (range 68–148), mean blood loss was 30 cc (range 0–60) and 12 patients ended with no measurable blood loss. The operative time was significantly shorter for 2 patients undergoing primary repairs: 68 and 75 min, respectively. All patients were discharged within 48 h of the surgery with 16 going home within 24 h. They all had a Foley catheter in place for at least 3 weeks when they were recalled to a follow-up appointment. In the outpatient clinic, a test was conducted by filling the bladder through the catheter at which all patients failed to detect a leak. Catheters were removed and patients discharged. No patients experienced recurrence at 3 years follow-up and no patients returned to our attention since.

## 4. Discussion

### 4.1. Summary of the Results

In our observational study, we demonstrated the safety and efficacy of our new technique, combined vaginal–laparoscopic repair (VLR), for the treatment of primary and recurrent VVF. We described the technique step by step together with detailed drawings to make the technique clear and simple for all clinicians involved in the treatment of this serious complication. In all our patients, the repairing was effective despite the mean large size of the VVF; all operations ended with the repairs being watertight. We had no intra-operative and post-operative complications with any of the patients that were discharged within 48 h of the surgery. Finally, at 36 months follow-up, no recurrences were reported.

### 4.2. Interpretation of the Results

In the Western world, VVF are the consequence of an iatrogenic injury occurring during surgery. Hysterectomy is the operation most frequently associated with VVF. Recently, a survey on data provided in England has worryingly demonstrated an increased rate [9]. However, the latter data are not confirmed in other countries [10]. VVF is an uncommon complication of surgery yet very stressful for patients and surgeons. The former are unprepared for such complications and suffer social and personal repercussions. The latter are likewise surprised by this rare occurrence and often lack the expertise to treat it. Therefore, there is an element of frustration because they caused a rare complication which is unlikely to heal and needs the intervention of other surgeons to be repaired. VVF demands treatment with a high chance of success; that is what patients and colleagues expect [11,12]. Over the years, alongside the success rate, surgery has aimed at reducing invasiveness and trauma. This occurred by preferring the extravesical to the trans-vesical technique and, in the last 15 years, by developing laparoscopic and robotic VVF repair [13,14]. The surgical route has been debated for years; a recent systematic review and meta-analysis reported a success rate of about 90% independently from surgical approach [1] As no clinical trial has been conducted, the choice of the physician is led by personal experience. However, all case series and reviews agree that the success rate of the VVF repair is highest at the first attempt and declines progressively in case of recurrent or persistent VVF [15,16]. While many reports are available on primary VVF repair, very few exist on patients with persistent VVF failing previous surgical attempts. In our series, with the exception of two patients who experienced complications from our own operations and had primary repair, all patients were referred from other hospitals following multiple failed repairs. We believe that in these patients, the VVF tract and the surrounding tissue need to be fully excised and cannot be used to repair the VVF. The main hindrances to the success of a repair are the dripping of urine on the vaginal tissue and the lack of satisfactory blood supply. Once the urine has excavated the VVF tract, the latter is covered by epithelium which facilitates the passage of urine and precludes the re-vascularization of the area. In addition, repeated surgical attempts will create further adhesions and fibrotic changes. Vesical uroepithelium, under normal vascular conditions, has phenomenal regenerative capacity and when damaged can heal within days [17]. Hence, the excision of the entire VVF tract, the vaginal and the vesical epithelium will eliminate inflamed, fibrotic, necrotic and ischemic tissue from healthy urothelium. This concept is even more valid in delayed VVF caused by use of diathermy and subsequent ischemia. Healthy uroepithelium, just as the vaginal epithelium, will heal rapidly irrespective of the size of the excision. We strongly advise to aim for sound margins well away from the VVF tract even when the excised area may seem too large to heal. The only limit to the excision must be the ureteric orifices which have to be clearly visualised and spared from the excision and the repair.

We want to underline the usefulness of a Foley catheter placed in VVF before the start of the surgical procedure. The choice of the catheter size is the largest fitting and it depends from the width of VVF. The use of Foley has several benefits that include the correct identification of vaginal part of VVF and its distance from the vaginal vault; at this point, VVF can be easily demarcated circumferentially by monopolar coagulation, leaving at least 1 cm of healthy tissue. Other benefits are related to the possibility of applying an appropriate traction to help the dissection and guarantee the exposure of sufficient tissue to suture the margin (essential in achieving a successful outcome in all patients with persistent VVF). This last concept is especially important during laparoscopic time for the correct resection of vaginal and bladder part of the VVF.

### 4.3. Comparison to Existing Literature

Surgical management of VVF has evolved considerably in developed countries. Despite this, there does not seem to be an approach, whether vaginal or abdominal, open or laparoscopic, flap or no flap, that is uniformly superior [3]. Moreover, existing literature regarding laparoscopic/robotic minimally invasive treatment of VVF is poor. No randomized controlled trials have been published. Despite these limitations, the demonstrated advantages regarding minimally invasive approaches are associated with less surgical trauma, shorter convalescence, and lower morbidity [3]. The recent systematic review and meta-analysis by Miklos et al. analyzing 44 published papers found that the overall success rate of laparoscopic repair was 80–100%; transperitoneal extravesical VVF repair has cure rates similar to the traditional transvesical approach; finally, there was no statistical difference in success rates of VVF repair with different number of layers in the fistula closure or with use of interposition flaps. Limitation of these results are related to the quality of existing literature: only case report/case series and few retrospective studies with less than 50 patients included; often no detailed description of surgical technique [7].

Concerning possible advantages of minimal approaches compared to the standard vaginal and abdominal route, a meta-analysis by Bodner-Adler et al. was recently published [8]. Authors included 107 papers and 1379 patients with VVF surgically treated. The transvaginal approach was performed in the majority of patients (39%), followed by a transabdominal/transvesical route (36%) and only 15% of patients were treated by laparoscopic/robotic approach (207 patients). Interestingly, authors reported a slightly better success rate by minimally invasive approaches (98.87% of success rate versus 97.05% for transabdominal/transvesicial route and 93.82% for transvaginal route). Moreover, comparing the use or not of an interposition flap, they reported no differences [8].

### 4.4. Strengths and Limitations

The main limitations of our study were certainly related to the small number of patients included and its retrospective design. However, our sample size is line with other published series and less than in the few retrospective studies which all included less than 50 patients [7]. Neither a prospective nor randomized trial has been published. Another limitation is related to the lack of a control group for comparing the equality or the superiority of our proposed technique. In addition, on this point, the literature is scarce with few comparative series published [7]; however, it is interesting that a recent meta-analysis reported a slightly higher success rate for a minimally invasive laparoscopic approach compared to the abdominal and vaginal route [8]. To account for the small numbers, it must be reminded that, luckily, VVF are rare events. 

The strength of our paper is certainly related to the rigorous surgical methods applied, standardized for each patient included. We described the technique step by step, together with detailed drawings, in order to make it easy to understand and reproducible to allow external validation.

### 4.5. Conclusions

In our study, we demonstrated the safety and efficacy of vaginal-laparoscopic repair (VLR) for the treatment of primary and recurrent VVF. This is the first report focused on persistent VVF following multiple failed repairs. The technique described is easily reproducible and has proved highly successful, in respect of a consistent protocol. It is an initial experience and needs confirmation on a larger number of patients.

## Figures and Tables

**Table 1:** Patient’s characteristics (*n* = 24) and previous surgery.

AGE	
Median, years	43.2 (SD 9.53)
Range, years	32–58
**WHO performance status (%)**	
0	(83.4%)
1	(16.6%)
2	0
**Body Mass Index (kg/m^2^)**	
Median (range)	27 (18–34)
**Previous Surgery**	
Number, (rate)	24 (100%)
**Surgery Causing VVF (Type and number)**	
Hysterectomy	18
Hysterectomy + lymphadenectomy	3
Cesarean section	3
**Attempted Repairs of VVF before VLR,** **Number, mean (range)** **and type, number**	
Number of attempts	2 (2–5)
Vaginal route	20
Laparoscopy	12
Laparotomy	10
Mixed route	14

**Table 2:** Surgical details and post-operative outcome.

Features	
Operative time (min)	108.3 (range 68–148)
Blood Loss (cc)	30 (range 0–60)
Hospitalization time (h)	36 (range 24–48)
Recurrence (3 years FU)	None

**Legenda:** Continuous variables are expressed as mean (range); FU: follow-up.

## Data Availability

Not applicable.
